# Fate of polycyclic aromatic hydrocarbons in the phytoremediation of different hydrocarbon contaminated soils with cotton, ryegrass, tall fescue, and wheat

**DOI:** 10.3389/fpls.2025.1550234

**Published:** 2025-04-22

**Authors:** Yunmin Zeng, Shijie Wang, Fan Huang, Qiang Luo, Bing Ren, Mohamed F. Abo El-Maati, Ahmed H. El-Sappah

**Affiliations:** ^1^ Faculty of Quality Management and Inspection & Quarantine, Yibin University, Yibin, China; ^2^ Key Laboratory of Treatment for Special Wastewater of Sichuan Province Higher Education System, College of Chemistry and Materials Science, Sichuan Normal University, Chengdu, China; ^3^ Key Laboratory of Urban Water Supply, Water Saving and Water Environment Governance in the Yangtze River Delta of Ministry of Water Resources, Tongji University, Shanghai, China; ^4^ Beijing Municipal Research Institute of Eco-Environmental Protection, Beijing, China; ^5^ Sichuan Tongyi Environmental Science & Technology Group Co., Ltd, Yibin, China; ^6^ Department of Biochemistry, Faculty of Agriculture, Zagazig University, Zagazig, Egypt; ^7^ School of Agriculture, Forestry and Food Engineering, Yibin University, Yibin, Sichuan, China; ^8^ Department of Genetics, Faculty of Agriculture, Zagazig University, Zagazig, Egypt

**Keywords:** aged oily sludge, petroleum contaminated soil, phytoremediation, polycyclic aromatic hydrocarbons, transfer calculation, microorganisms

## Abstract

**Introduction:**

Phytoremediation is a promising strategy for cleaning up polycyclic aromatic hydrocarbon (PAH)-contaminated soils. This study investigated the effectiveness of four plant species—cotton, ryegrass, tall fescue, and wheat—in enhancing PAH removal from soils contaminated with diesel oil, PAHs, and aged oily sludge.

**Methods:**

Aged oily sludge-contaminated soil was artificially prepared, and the selected plants were cultivated in different hydrocarbon-contaminated soils (diesel oil, PAHs, and oily sludge). The fate of PAHs was analyzed by measuring their distribution in rhizospheric soil and plant tissues. Root concentration factors (RCFs) and transpiration stream concentration factors (TSCFs) were used to evaluate PAH translocation and accumulation in plant tissues and their interactions with the rhizosphere.

**Results:**

The study demonstrated that plants enhanced PAH removal by 20%–80%, with wheat showing the highest efficiency. PAH removal was generally more effective in oily sludge-contaminated soil than in diesel oil or PAH-contaminated soil. Plant uptake of PAHs accounted for 2%–10% of total removal and exhibited a strong linear correlation with root weight. RCFs were linearly correlated with LogKow (3–6), indicating that the four plant species did not significantly concentrate PAHs in their roots.

**Discussion:**

The findings confirm the potential of phytoremediation for PAH-contaminated soils, particularly using wheat as an effective species. The low RCFs and TSCFs suggest that PAH uptake was limited, implying that rhizodegradation and microbial interactions may play a more critical role than direct plant accumulation. This study supports phytoremediation as a cost-effective and eco-friendly alternative to conventional soil remediation methods, reducing economic and environmental burdens.

## 1 Introduction

Recently, contaminated soils with PAHs have been extensively studied due to their persistence and toxic, mutagenic, and carcinogenic effects, posing a high risk to ecosystems and human health. Many methods have been used to clean up PAHs-contaminated soil sites (Kothiyal et al., 2022). While total petroleum hydrocarbon (TPH) is used to characterize the contamination in soil, polycyclic aromatic hydrocarbons (PAH) are often used to assess risk to the environment and/or humans (Okparanma and Mouazen, 2013; Kaur and Sharma, 2020). In the U.S. EPA, 16 PAHs have been used to assess the efficacy of treatments to remove PAHs for several years (Okparanma and Mouazen, 2013; Bojes and Pope, 2007), after which a comprehensive list of PAHs were used (Yang et al., 2021, Yang et al., 2017; Park et al., 2018; Ellickson et al., 2017).

Plant-promoted biodegradation and uptake of organic compounds from polluted soils has wide implications for site remediation since the process is critical for understanding contaminant exposure pathways involving vegetation, mainly due to its cost-effective, ecologically friendly, applied *in situ* and less disturbance of soil matrix, another crucial advantage was that phytoremediation usually resulted in complete mineralization of the pollutant (Feng et al., 2017). Compared with the physical and chemical methods. Using green plants, including herbs and woody species, which can remove, uptake, or render harmless, various environmental contaminants like heavy metals, organic compounds, and radioactive compounds in soil, phytoremediation is proposed as a relatively recent technology with sustainable costs and is a most significant advance to physical and chemical methods (Ali et al., 2013; Tahir et al., 2016). In the phytodegradation process, thanks to enzymes, organic contaminants are absorbed and involved in the metabolism of the plants; as a result, they are degraded. The combined application of sodium nitroprusside and melatonin can markedly improve drought tolerance in maize, promoting enhanced plant growth and increasing its capacity to absorb organic contaminants (Ullah et al., 2025, Ullah et al., 2024). Dehalogenase, peroxidase, nitroreductase, nitrilase, and phosphatase were found to play roles in this process (Deng and Cao, 2017; Winquist et al., 2014; Perez et al., 2021). Besides, they also could be incorporated into the plant tissues (Ali et al., 2013; Van Oosten and Maggio, 2015). The most significant advantage of phytoremediation is that the contaminant can be transformed into a less toxic substance. Through phytovolatilization, pollutions, mainly organic, were taken into the air; however, pollutions still exist (San Miguel et al., 2013; Yu and Gu, 2006; Ali et al., 2013; Van Oosten and Maggio, 2015). Most pollutants in the atmosphere are degraded through photooxidation, washed back to the surface by rain, or removed by other natural processes.

The distribution of PAHs in plants was applied to describe the uptake and translocation of organic compounds; the factors that worked in these processes were found to be their chemical characters like octanol/water partition coefficient (Kow), water solubility, hydrophobicity (lipophilicity), polarity and molecular weight (Cofield et al., 2007; Mamirova et al., 2021). These all indirectly reflect the solubility of organic compounds; thus, researchers have focused on correlations between partition factors and chemical properties that express relative solubility, such as K_ow_. (Zhang et al., 2017) found that The translocation of PAHs in maize tissues has a positive relationship with log Kow less than 4.5, while negatively correlated otherwise. The vertical PAHs distribution in soils indicated that 2–3 rings PAHs with a low octanol-water partition coefficient (log Kow < 4.5) were easier to transport in soils, causing a great potential risk of immigrating to the groundwater. More hydrophobic compounds were firmly bound to root surfaces or partitioned into root solids, resulting in less translocation within the plant. Mandal (Mandal et al., 2018; Wang et al., 2016) successfully remediated oil-contaminated saline-alkali soil using nine plants, including cotton, ryegrass, tall fescue, and wheat. (Li et al., 2023) selected 20 types of plant seeds for petroleum hydrocarbon-contaminated soil remediation. They conducted degradation experiments on petroleum hydrocarbons using seeds of five plants, such as cotton and peanuts, which are suitable for growth and have a high germination rate. The degradation rate reached 38.9% in 70 days.

Interactions exist among PAHs, which play a role in their phytoremediation process, ultimately affecting their efficacy (Ahmad et al., 2024; Liu et al., 2023). While most previous studies have predominantly focused on the biodegradation of individual PAHs, our project sought to explore the broader dynamics of PAHs biodegradation by investigating the effects of different PAHs sources and plant species on the remediation process. This project studied the effects of PAHs biodegradation of the different PAHs resources (e.g., aged oily sludge, diesel oil, and PAHs solution) and plant species (e.g., cotton, ryegrass, and tall fescue). The objectives of this project were designed to provide a comprehensive understanding of the mechanisms and outcomes of PAHs biodegradation in complex environmental systems, which were as follows: (1) Quantify and compare the biodegradation efficiency of polycyclic aromatic hydrocarbons in different experimental groups and determine the optimal conditions for removing polycyclic aromatic hydrocarbons. (2) Evaluate the transfer ability of polycyclic aromatic hydrocarbons between different PAHs contaminated soil matrices and plant tissues and reveal PAHs’ absorption, transport, and accumulation patterns within plant systems and the rhizospheric soil.

## 2 Materials and methods

### 2.1 Soil preparation and phytoremediation

In this study, the peat (**Table 1**) from Jilin province was used to (1) greatly enhance the physical structure of contaminated soil, mainly in the aspects of porous ratio, water holding capacity, and aggregation due to the greater volume of peat; (2) increase the nutrient content as a slow-release green manure due to containing a considerable variety of nitrogen, phosphorous and potassium at the values of 1.49% N, 0.12% P and 1.26% K; (3) modify the pH and salinity since the peat has a large content of humic matters, and subsequently can buffer or stabilize soil pH; (4) improve the biodegradation activity largely based on the presence of organic matter and microorganisms including bacteria, protozoa, actinomycetes, and fungi. In summary, adding peat was expected to have an immediate and long-term positive impact on the phytoremediation of aged oily sludge-contaminated soil.

**Table 1:** Physical-chemical properties of the added peat.

Added Peat	Total N (*m*%) 1.49	Available N (mg/kg) 844 ± 37.84	Ash Content (*m*%) 15.4
	Total P (*m*%) 0.12	Available P (mg/kg) 167 ± 7.37	Specific Gravity 0.84
	Total K (*m*%) 1.26	Available K (*m*%) 0.72	Coarse Protein (*m*%) 9.31

All indicators were measured three times, and the experimental data were averaged after removing outliers.

The oily sludge was sampled from the Shengli Oil Field of Shandong province in China with an initial TPH content of 12.36 ± 0.04%. The background soil was found to contain 0.02% TPH, a concentration that falls below the quantification limit of 24 mg/kg. The diesel oil and PAHs contaminated soils were prepared by mixing diesel oil and PAHs solution with pristine soils and subsequently aged for 6 months to result in only desorption and volatility-resistant fraction. The soil samples were prepared by mixing with 10% peat and background soil to reach the final TPH of 6.31 ± 0.06% (aged oily sludge contaminated soil), 3.05 ± 0.05% (diesel oil contaminated soil) and 0.16 ± 0.03% (PAH contaminated soil), respectively. No PAHs were detected in the background soil throughout this study. Determine the concentration of total petroleum hydrocarbons using gas chromatography. The detection limit of this method for total petroleum hydrocarbons is 6mg/kg, and the limits of quantification is 24mg/kg.

The contaminated soil (2000 g per pot) was added to 15 cm diameter, 20 cm depth, and 3.0 L volume figuline pots. The soils were allowed to equilibrate for 7 d at field moisture before the introduction of plants. The pots were sealed (i.e., no drainage holes) to avoid the pollutant leaching. Then they were sowed at a density of 2.9-4.5g/m^2^, 2.3-3.0g/m^2^, 15-20g/m^2^ for cotton (*Gossypium hirsutum*), ryegrass (*Lolium multiflorum Lam.*) and tall fescue (*Festuca arundinacea*), respectively. All of the 72 pots (each treatment- plant species for aged oily sludge contaminated soil, diesel oil contaminated soil, and PAHs contaminated soil was established with six replicates, while the treatment without plants (served as natural attenuation) was placed in a greenhouse in Beijing, with a temperature of 20-25°C and soil moisture in a range of 42-67%.

Based on field investigations and preliminary experimental findings of dominant plant species around the contaminated site, ryegrass, tall fescue, cotton, and wheat were identified as having significant resistance and removal potential for PAHs. Consequently, these four plant species were selected as the target plants for this study. According to the preliminary experiment results, the target plants selected for the experiment can better reflect the stress effects of pollutants on plants within a 5-month.After 5 months of phytoremediation, the rhizospheric soil samples were collected, air dried, and passed through a 0.6-cm sieve to remove the root residues before -20°C store, while the plant tissue samples were collected separately to represent aboveground and root tissues, and then the plant samples were air-dried, weighted, crushed, bagged, and stored.

### 2.2 PAHs analysis and data processing

Extraction of PAHs was performed with 10 g samples by Soxhlet using a 1:1 dichloromethane and acetone mixture for 12 h. Extracts were pretreated through a cleanup column with silica gel and anhydrous sodium sulfate. Then, they were condensed by evaporation under a stream of nitrogen and a 40 °C water bath; finally, the extracts dissolved in 1ml Hexane.

The concentrations and profiles of PAHs were analyzed by Agilent 7890A gas chromatography equipped with a 5975C mass detector. The capillary column used was a DB-5 (30m×0.32mm i.d.×0.25m film thickness). The initial column temperature of 80°C for 1 min, 15°C/min to 265°C for 1 min, and then 2.5°C/min to 300°C for 5 min. The temperatures of the injector and detector were at 300°C and 280°C. The carrier gas was nitrogen at a constant 1.0 ml/min flow rate. Identification and quantification of 16 PAH compounds were based on SIM scan and internal standards (Naphthalene-d8, Acenaphthene-d10, Phenanthraene -d10, Chrysene-d12, Perysene-d12), and the same extraction determined the procedural blank. The system monitoring recovery rates of Nitrobenzene-d5 and 4-Terphenyl-d14 were 95% and 90%, respectively. In this study, the lowest detection for soil and plant samples were 0.01 μg/g and 0.05 μg/g, respectively.

The formula gave the percentage of PAHs removal in rhizospheric soil (D%): D%= 100%×(M_i_−M_f_)/M_i_, in which M_i_ was the initial concentration of PAHs; M_f_ was the final concentration in each treatment after the phytoremediation.

Root concentration factors (RCFs) have been defined as a simple partition coefficient between the total plant tissue and the external soil condition, while the transpiration stream concentration factors (TSCFs) were defined as the partition coefficient between the tissues underground and aboveground.


$$RCF\text{s}=\frac{Cp}{Cs}\;;\;TSCFs=\frac{Cu}{Cr}$$

C*p* and C*s* stand for the concentration of ∑PAHs in plants and soil, respectively.

C*u* and Cr C*r* represent the concentration of ∑PAHs in the plant tissues of roots and above ground, respectively.

### 2.3 Statistical methods

All indicators were measured three times, and the experimental data were averaged after removing outliers. Microsoft Excel (version 2019) software was used for statistics and calculation of experimental data. SPSS (version 26.0) was used for data correlation analysis, and experimental drawing was completed by Origin software (version 2024).

## 3 Results

### 3.1 The physical-chemical properties of soil samples

It is advantageous to use multiple techniques or processes to accelerate remediation kinetics and increase plant and microbial biomass for practical and effective remediation of various environmental contaminants. Therefore, the peat was added to the polluted soils. The physical-chemical properties of the background soil, added peat, and aged oily sludge (which was sampled from Shengli Oil Field) were detected according to the Chinese national standard, which includes pH, CEC, organic matter, bioavailable N/P/K, heavy metal content, and so on.

As shown in **Table 2**, the aged oily sludge was composed of 23.93% TPH with 9.57% saturates, 7.18% PAHs, 4.79% resins, and 2.40% asphaltenes, of which the saturates and PAHs fractions can be biodegraded. Still, the resins and asphaltenes fractions are traditionally considered recalcitrant to microbial alteration and increase the polar fraction, viscosity, oil density, sulfur content, and acidity. It was accepted that the available nutrient was one of the limitations for bioremediation of organic contaminated soils; as shown in **Table 2**, the content of N/P was at a relatively low level of 15.00 and 78.00 mg/kg.

**Table 2:** Physical-chemical properties of the aged oily sludge sampled.

Type	pH	Specific Gravity	TOC (%)		Salinity (g/kg)	CEC (cmol/kg)	TN (mg/kg)	TP (mg/kg)	TK (mg/kg)
Arenarious Loam	8.60	2.74	2.33		63.04	5.44 ± 0.17	15.00 ± 1.57	78.00 ± 9.09	1690.00 ± 191.78
	Clay (<0.002mm)%				Slit (0.02-0.002mm)%		Sand (2-0.02mm)%		
	13.71				29.62		56.67		
Moisture (*m*%)	Ash Content (*m*%)				Volatile Content (*m*%)		Fixed Carbon (*m*%)		
20.35	62.13				32.14		6.21		
TPH (*m*%)	PAHs (*m*%)			Resins (*m*%)	Saturates (*m*%)		Asphaltene (*m*%)		
23.93	7.18			4.79	9.57		2.40		
As (mg/kg)	Hg (mg/kg)			Cu (mg/kg)	Cd (mg/kg)	Cr (mg/kg)	Zn (mg/kg)		Pb (mg/kg)
8.81 ± 0.95	0.02 ± 0.0013			17.52 ± 2.35	0.05 ± 0.008	42.32 ± 6.16	64.71 ± 4.79		26.21 ± 0.66

All indicators were measured three times, and the experimental data were averaged after removing outliers.

### 3.2 The removal of PAHs in rhizospheric soils of ryegrass, tall fescue and cotton

The initial ∑PAHs of contaminated soils were 297.79μg/g, 228.44μg/g, and 8.63μg/g for PAHs contaminated soil (blue column), diesel oil-contaminated soil (red column) and aged oily sludge-contaminated soil (white column), respectively. The experimental replication number is n=6. The CK experimental group involved soil with the same pollutants and concentrations, amended with an equivalent amount of the peat, but without planting any vegetation, while maintaining identical watering conditions to assess the natural microbial degradation of PAHs. CK represents the natural attenuation assay.

**Figure 1:**
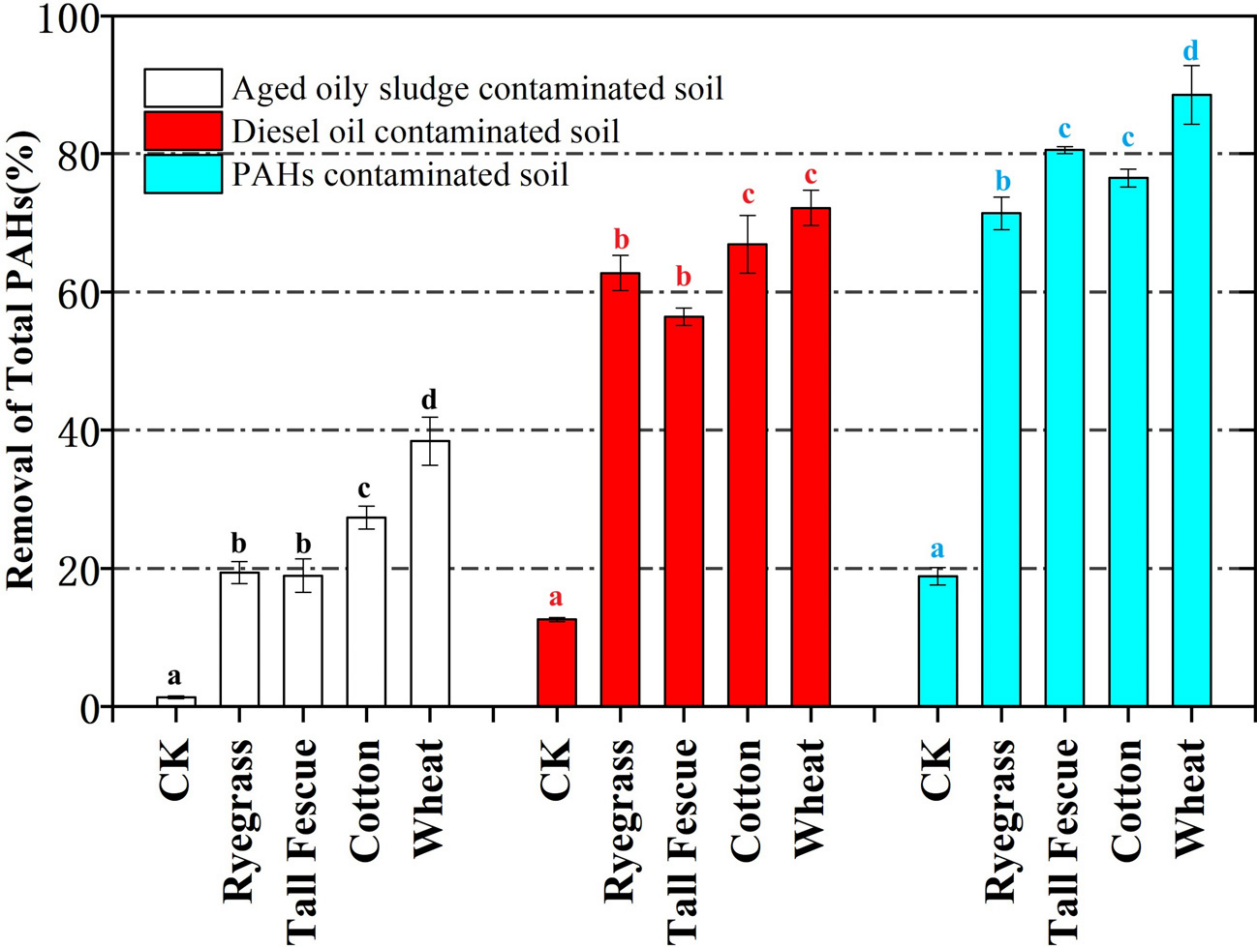
Total PAHs removal (in m%) in rhizospheric soils contaminated with aged oily sludge, diesel oil, and PAHs after a 5-month phytoremediation period using ryegrass, tall fescue, cotton, and wheat. Significant differences between the tested plants and the control were indicated by different letters (a, b, c and d) (P < 0.05, Student's t test).

**Figure 1** demonstrates the efficacy of phytoremediation in enhancing the removal of polycyclic aromatic hydrocarbons (PAHs) from contaminated soils. As illustrated in **Figure 1**, the removal rate of PAHs in the CK (control) experimental group was notably low, all below 20%, indicating that natural attenuation alone is insufficient for effective remediation. In contrast, the experimental groups with planted vegetation achieved PAHs removal rates ranging from 18.97% to 88.53%, all significantly higher than the CK group, underscoring the critical role of phytoremediation in improving PAHs removal efficiency. Among all plant species, wheat exhibited the highest PAHs removal rate, reaching 88.53%, consistently outperforming other plants. This suggests that wheat possesses a superior capacity for PAHs uptake, translocation, or stimulation of rhizosphere microbial degradation, establishing it as a dominant species for the phytoremediation of PAHs-contaminated soils. The efficiency of PAHs removal varies considerably based on the type of contamination. Among the three soil types tested, PAHs contaminated soil demonstrated the highest PAHs removal efficiency, with all plant species showing significant remediation effects. In contrast, aged oily sludge-contaminated soil exhibited the lowest PAHs removal efficiency, with only 38.37% removal in the wheat group. This lower efficiency may be attributed to the complex composition and high toxicity of aged oily sludge, which could generate more toxic intermediate products or exhibit reduced bioavailability during aging.

Plant uptake of total PAHs was 2.03% -11.27%. Therefore, this study provided evidence that the plant root uptake of total PAHs was not a significant removal pathway; the loss of PAHs is likely due to rhizosphere biodegradation and the strong association of PAHs with organic particles (Mesa-Marin et al., 2019). As shown in **Figure 1**, the highest uptake of PAHs appeared in the wheat-mediated phytoremediation assay of aged oily sludge (1.27%). The plant uptake of total PAHs varied with the type of contaminated soil and phytoremedial plants: its value for the aged oily sludge contaminated soil was about 2-6 times and 10-40 times higher than that for the diesel oil contaminated soil and PAHs contaminated soil, which can be attributed to the properties of contaminated soil such as the type and concentration of contaminants.

### 3.3 The distribution and transfer of PAHs in ryegrass, tall fescue, and cotton

**Figure 2:**
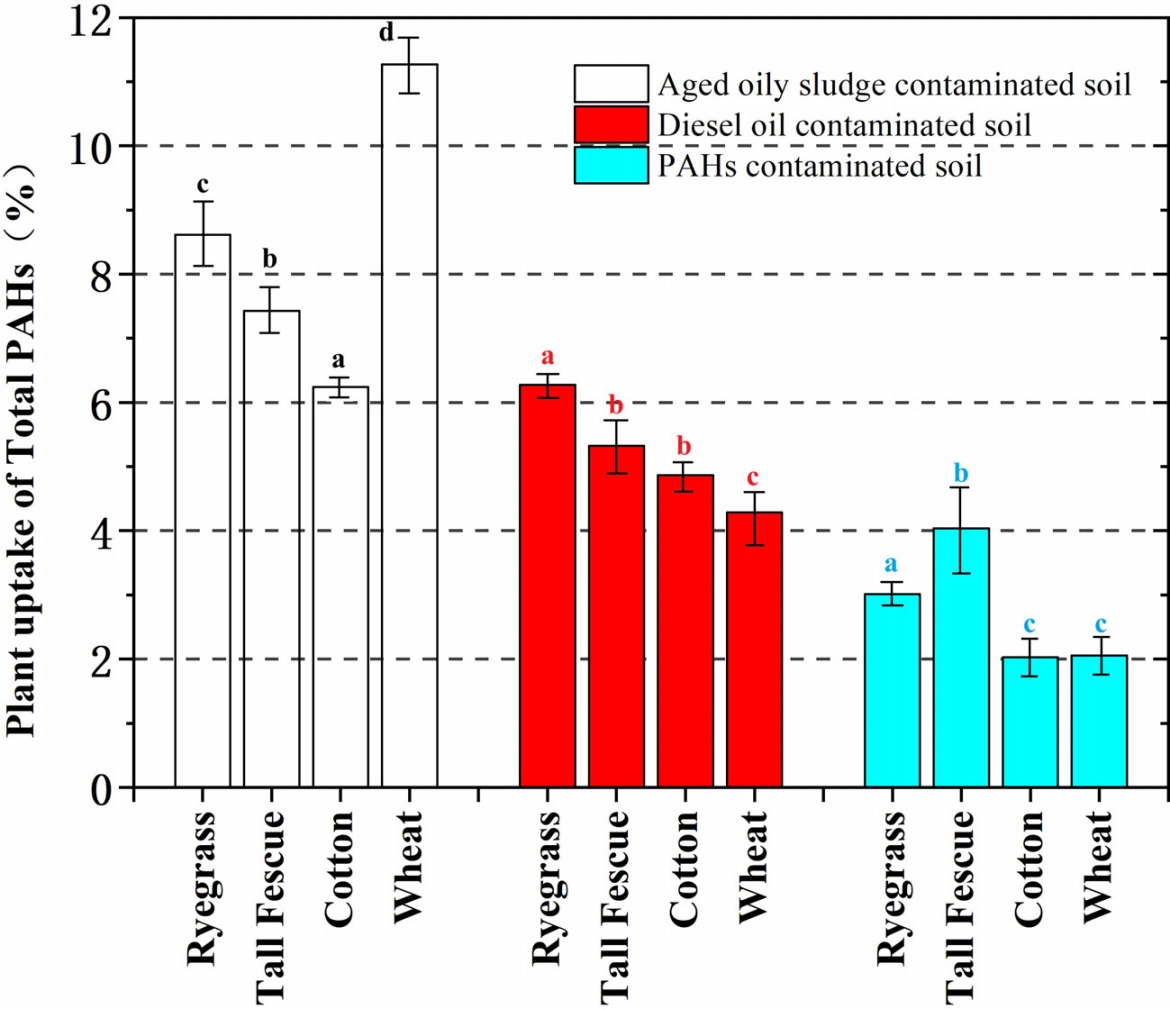
Plant uptake of total PAHs by ryegrass, tall fescue, cotton, and wheat in soils contaminated with aged oily sludge, diesel oil, and PAHs. Significant differences between the tested plants and the control were indicated by different letters (a, b, c and d) (P < 0.05, Student's t test).

As shown in **Figure 2**, the percentage of PAHs uptake is in the range of 2.03%-11.27%, and the uptake of PAHs was in the order of aged oily sludge contaminated soil (6.24%-11.27%), diesel oil contaminated soil (4.27%-6.27%), and PAHs contaminated soil (2.03%-4.34%). The observed result in this study was probably due to the toxicity induced by different concentrations of PAHs, which may limit plant growth and subsequently influence the uptake of PAHs (Feng et al., 2017).

**Figure 3:**
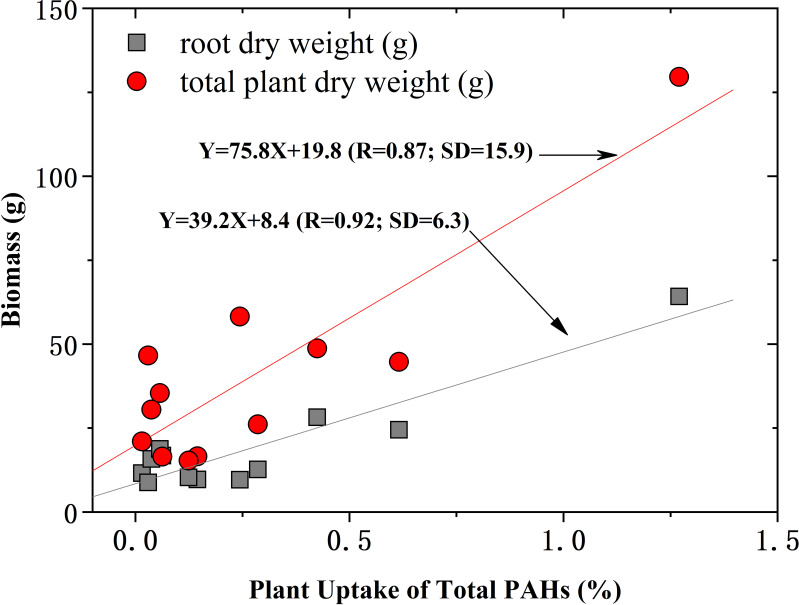
The relationship of plant uptake with biomass of root and total plant weight.

As shown in **Figure 3** and **Supplementary Table S1**, the linear regression between the plant uptake of PAHs and biomass of total plant and root was observed with correlation coefficients of 0.87 and 0.92, suggesting that the location of the root was the pool for accumulating PAHs from the soil. The distribution of PAHs in different plant parts is always determined and evaluated by the root concentration factors (RCFs) and transpiration stream concentration factors (TSCFs).

**Figure 4:**
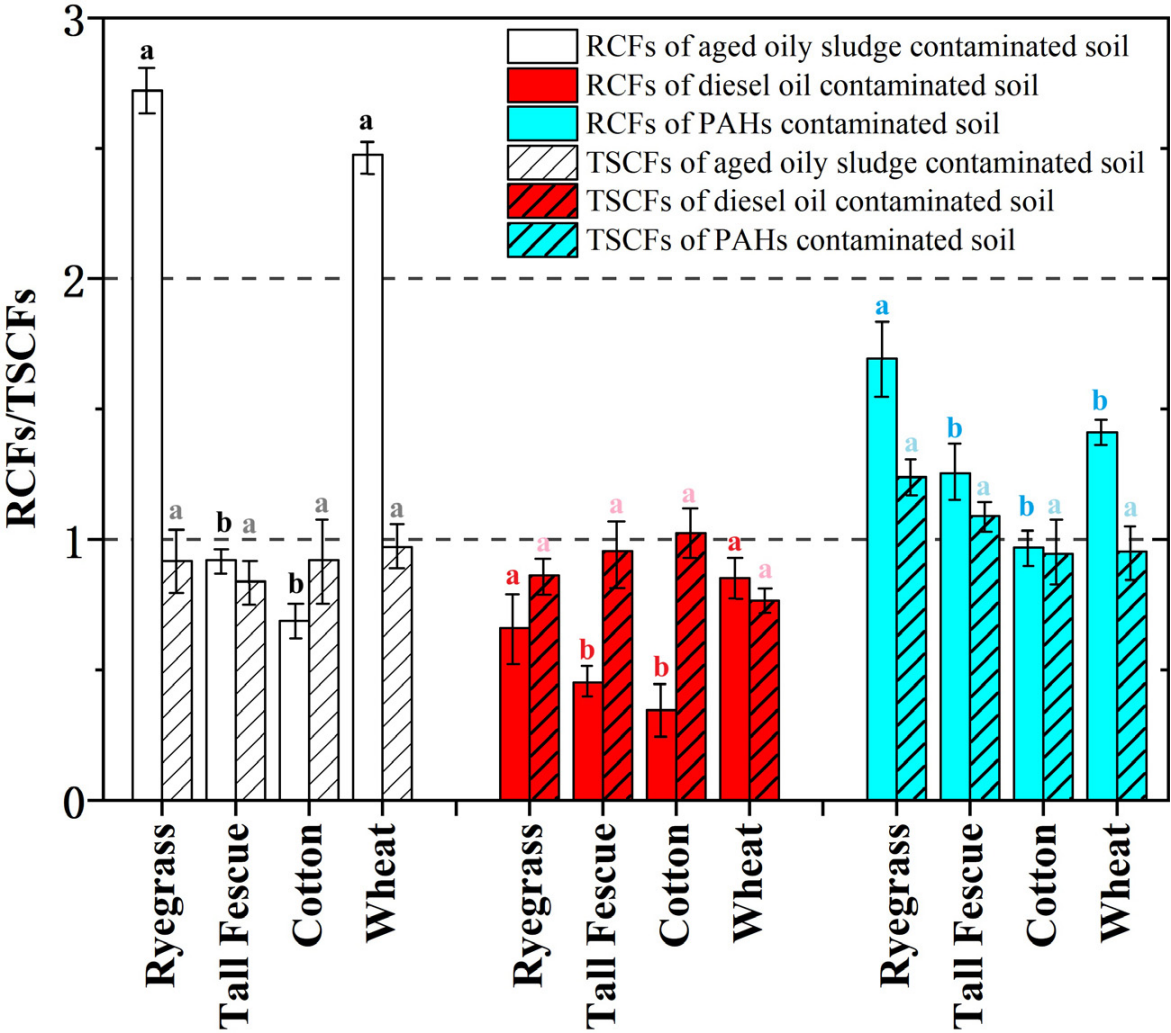
Root concentration factors (RCFs, blank legends) and transpiration stream concentration factors (TSCFs, sparse twill legends) of ∑PAHs after 5 months phytoremediation. Significant differences between the tested plants and the control were indicated by different letters (a and b) (P < 0.05, Student's t test).

As shown in **Figure 4**, the values of RCFs varied with the plant species and types of contaminated soils; in aged oily sludge-contaminated soil, ryegrass and wheat had the higher ability of root concentration with RCFs of 2.72 and 2.48, respectively, but it did not reach the standard for screening the hyperaccumulator. The rest of the RCFs had no significant difference. In addition, the values of RCFs in the diesel oil-contaminated soils were generally lower than that in the PAHs-contaminated soils and aged oily sludge-contaminated soils; this may be due to the enhanced cometabolism of PAHs with the saturated hydrocarbons, and the toxicity of higher PAHs concentration may be the reason resulting in lower values of RCFs in PAHs contaminated soils. Results indicate that annual ryegrass can establish and survive in anthracene-contaminated soil at a concentration of 100 mg/kg dry soil and showed no outward signs of phytotoxicity. There was no significant effect of phenanthrene (contaminated at 1000 mg/kg) on the root-soot biomass of ryegrass compared to the uncontaminated soil. It was also noted that the values of TSCFs showed no significant difference among different remediation ways, indicating that the PAHs translocation from soil to the various plant sections (e.g., root, stem, leaf, fruit, seed, etc.) was primarily determined by the transmembrane transport, and the driving force for PAHs distribution in different plant sections was similar among the ryegrass, cotton, wheat, and tall fescue.

### 3.4 Correlation analysis of the RCFs and Kow of 16 PAHs

**Figure 5:**
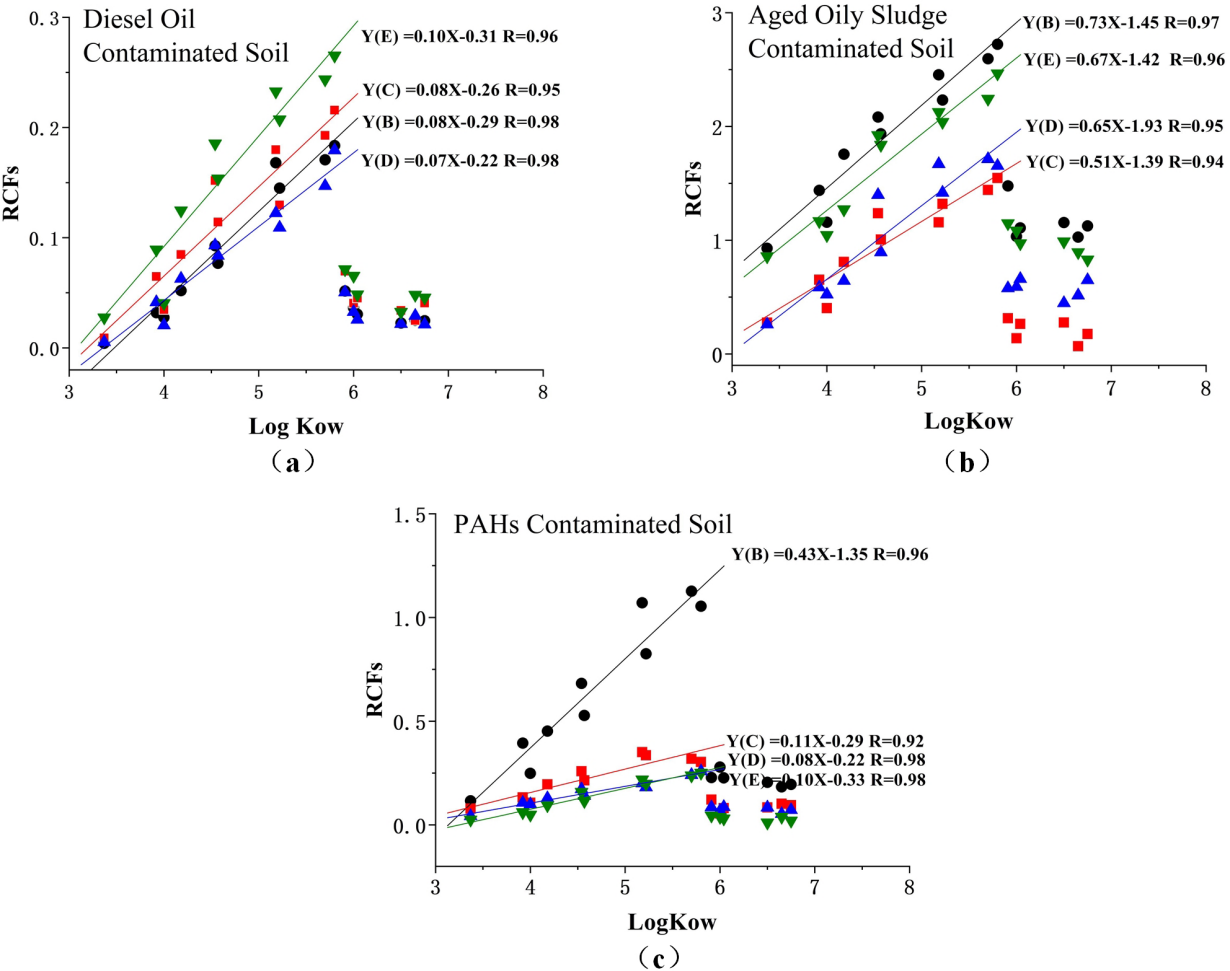
Relationship between RCFs of 16 priority PAHs in the diesel oil contaminated soil **(a)**, aged oily sludge contaminated soil **(b)**, and PAHs contaminated soil **(c)**. Y-phytoremediation with (B) ryegrass, (C) tall fescue, (D) cotton, (E) wheat.

As shown in **Figure 5**, the relationship between the RCFs of 16 priority PAHs and LogKow tended to be linear. However, only for the logKow between 3 and 6, it suggested that the higher values of Kow had higher uptake potential by plant tissue. The different values of RCFs depended on the chemical properties and related to the plant species and the soil types. However, analyzing the complexity of this relationship requires more analysis of the conformational space of chemicals studied across the whole range of logKow values. As for the PAH compounds with logKow higher than 6, the RCFs values were not significantly different but relatively lower than the PAH compounds of logKow between approximately 3 and 6; this may be due to the higher size of molecule limiting its transmembrane.

## 4 Discussions

### 4.1 The influencing factors of the phytoremediation of aged oily sludge-contaminated soil

The added nutrients were necessary for the phytoremediation of aged oily sludge. In addition, the oily sludge sampled from Shengli Oil Field was 63.04 g/kg salinity and 8.60 pH value, which determined that the potential plant species for phytoremediation should be tolerant to salified and alkalinized soils. The oily sludge was arenarious loam type with 13.71% clay, 29.62% silt, and 56.67% sand, and the heavy metal determination revealed that their content of As, Hg, Cu, Cd, Cr, Zn, and Pb was not beyond the second category standard with respective to National Environmental Quality Standard for Soils (GB 15618-1995, China). Adding peat was expected to have an immediate and long-term positive impact on the phytoremediation of aged oily sludge-contaminated soil. Their source and distribution greatly influenced the biodegradation of PAHs.

### 4.2 The effects of plant species in PAHs removal mechanism and rhizosphere interactions

As shown in **Figure 1**, the removal of total PAHs varied with different contaminated soils and phytoremedial plants. Plants can enhance the phytoremediation efficiency of PAHs with 20–60% more PAHs removal than that in CK (natural attenuation). This result was consistent with other research (Alves et al., 2018; Ma et al., 2020; Johnson et al., 2005; Xu et al., 2014; Rasmussen and Olsen, 2004), and the significantly enhanced PAHs’ removals were probably attributed to the effects of the augmented rhizosphere microorganisms and/or uptake by plant tissues (Xu et al., 2014; Zelko et al., 2017; Liao et al., 2021). Plants secrete various compounds (e.g., organic acids, sugars, and proteins) into the soil, which can stimulate or inhibit microbial growth and activity. Bioaugmented phytoremediation using cowpea enhanced microbial growth, increased soil dehydrogenase and invertase activities, improved bacterial community diversity, and accelerated the rhizodegradation of total petroleum hydrocarbons (TPH) in oil-contaminated soil (Yang et al., 2022). The phytoremediation efficiency of PAHs was in the order of ryegrass, tall fescue, cotton, and wheat since plant species differ in PAHs removal mechanism and effects on rhizosphere microorganisms; furthermore, Yang proved that the ryegrass had a higher rhizosphere effect on the PAH removal than other plant species (Yang et al., 2007), and Merkl found that the biodegradation rate of PAHs was dependent on individual composition of plant exudates (Merkl et al., 2005). Guo et al (2017) indicated that ryegrass increased the degradation of PAHs by promoting bacteria diversity, increasing the abundance of total bacteria and PAH degraders, and stimulating PAH-ring hydroxylating dioxygenase (*PAH-RHDα*) genes expression.

### 4.3 The effects of soil characterization in PAHs removal mechanism

The survival and activity of microorganisms and the degradation efficiency of PAHs are significantly influenced by variations in soil types and their inherent properties. The removal of PAHs in aged oily sludge was the least even though its initial concentration of PAHs was the smallest among the three contaminated soils, suggesting that the characterization of contaminated soils may be the key influencing factor for the PAHs biodegradation (Ma et al., 2020). The tight binding and high competition with saturated hydrocarbons render the PAHs unavailable to some plants and(or) results in poor uptake for aged oily sludge and diesel oil-contaminated soils (Xu et al., 2014; Al Sbani et al., 2021).In this study, the fate of 16 PAHs (Bojes and Pope, 2007) in the rhizosphere was studied, which showed the capability of planted soil with ryegrass, tall fescue, cotton, and wheat in accelerating the PAHs dissipation, even including the 5-6 ring PAHs of dibenzo(a,h)anthracene and benzo (g, h, i)perylene. The disappearance of PAHs with three or more rings was mainly attributed to biotransformation or biodegradation (Mandal et al., 2018). However, the peat used in improving the soil conditions increases the organic matter content, potentially leading to increased sorption of contaminants and humification (incorporating a compound into organic matter). As a result, the increased dissipation of the PAHs in the rhizosphere may also be due to a decreased extractability of the PAHs with the formation of bound residues.

### 4.4 The effects of plant root morphological structure in PAHs removal mechanism

In this study, the plant species of ryegrass, tall fescue, and wheat had a large fibrous root, which has a deep, extensive fibrous root network, maximizing the root-soil contact and the influence of the rhizosphere. Due to the complex spatial structure of fibrous roots, the contact area between the roots and the soil was larger, which provided more colonizing space for microorganisms; besides, the complex network structure led to the densification of the whole space of the root system, thereby enhancing the interaction between microorganisms and pollutants (Liao et al., 2021). In addition, the developed root system made the soil structure crisp and loose, increasing the voids, which was also conducive to the circulation of air and water in the soil, providing more oxygen and energy for microorganisms, thereby reducing the content of pollutants in the soil (Liao et al., 2021; Oniosun et al., 2019; Chen et al., 2018; Wei et al., 2017).

Due to their strong adhesion, PAHs with strong hydrophobic properties were firmly adhered between roots and soil, so it wasn’t easy to transfer inside plants. Due to the strong water solubility of those chemicals (logKow<1.0), after being diluted by water, it isn’t easy to adsorb to plant roots and cannot be actively transported through plant membranes. Hydrophobic chemicals (logKow>3.5) are candidates for phytostabilization and/or rhizosphere biodegradation. In this study, the different RCFs of PAHs in plant tissue were also related to their biodegradation. The cometabolism capacity of 4-ring PAHs is positively correlated with biodegradable 2-3-ring PAHs, and after the reduction of the latter, plants released phenolic compounds (Jarujareet et al., 2019; Gonzalez-Costas et al., 2020) that acted as PAH analogs. Studies indicated that increasing the removal efficiency of aged TPH by plants was achieved by increasing the rhizosphere microorganisms because these microorganisms contain a bulk of hydrocarbon catabolic genes (Siciliano et al., 2003).

Before transpiration transfers the pollutant to other plant tissues, it must overcome the barriers created by detoxification and metabolism in the roots (Feng et al., 2017). In this process, glutathione-S-transferase was responsible for the binding reaction that plays a central role in plant detoxification. The third stage of phytobiotic metabolism involves storing and segregating soluble conjugates in the cell wall.

### 4.5 The potential applications of phytoremediation technology in addressing petroleum hydrocarbon-contaminated soils

The three plants studied effectively remove petroleum hydrocarbon pollutants such as diesel, polycyclic aromatic hydrocarbons, and oily sludge. This demonstrates that phytoremediation technology can address leakage pollution of petroleum hydrocarbons in product samples, purified samples, and solid waste samples. The remediation capabilities of these plants are applicable across diverse scenarios, including mining sites, transportation processes, petroleum derivative production facilities, and solid waste treatment stations.

The findings of this study provide valuable insights and references for applying phytoremediation in soils contaminated with PAHs and other petroleum hydrocarbons. Moreover, developing integrated remediation technologies, such as microbial-plant and chemical-plant approaches, is crucial to unlocking the full potential of phytoremediation and enhancing the removal efficiency of petroleum hydrocarbons. Similarly, addressing complex polluted sites is essential, as petroleum-contaminated areas often involve heavy metals. Plants also possess the ability to mitigate heavy metal accumulation. Cultivating plants for the remediation of petroleum hydrocarbon-heavy metal composite pollution can contribute significantly to advancing phytoremediation technology. In the future, with the continuous development of plant remediation technology and microbial engineering, research on the mechanisms and interactions of plants and rhizosphere microbial systems in degrading PAHs will continue to deepen. Biological, environmental remediation technology will be further improved and enhanced, providing more effective solutions for treating PAHs pollution.

## 5 Conclusion

Experiments have shown that the root position is the accumulation position of PAHs in soil, and the uptake of PAHs by plants has a linear relationship with the total plant and root biomass. The degradation efficiency of PAHs by phytoremediation was 10%-60% higher than that of CK, and the restoration efficiency was ryegrass, tall fescue, cotton, and wheat. The absorption of PAHs by plants is only 2.03%-11.27%; it is evident that the removal of PAHs in soil by phytoremediation is mainly achieved through the metabolism of hydrocarbons by root microorganisms. Plants with developed root systems, due to the complex heel system, not only provide many colonization spaces for microorganisms but also improve the mobility of air and water, thereby improving the degradation efficiency of PAHs by plants. The ability of plants to absorb and transport PAHs is related to their chemical properties. Among the 16 PAHs studied, those with strong hydrophobicity (logKow>3.5) are candidates for plant or microbial remediation.

## Data Availability

The original contributions presented in the study are included in the article/**Supplementary Material**, further inquiries can be directed to the corresponding author/s.
