# Supplementary material for: Fate of polycyclic aromatic hydrocarbons in the phytoremediation of different hydrocarbon contaminated soils with cotton, ryegrass, tall fescue, and wheat
Source: Front Plant Sci. 2025 Apr 22;16:1550234. doi: 10.3389/fpls.2025.1550234 (PMC12053178; doi:10.3389/fpls.2025.1550234)
Supplement: Supplementary file 1 [file DataSheet1.pdf]

Analysis Result

Sample status: Plant sample		Sample number<br>CAS	LOR	unit	SH110051<br>6-001	SH110051<br>6-002	SH110051<br>6-003	SH110051<br>6-004	SH110051<br>6-005
**多环芳烃 (Polycyclic aromatic hydrocarbons， PAHs)**									
萘	Naphthalene	91-20-3	0.05	mg/kg	<0.05	<0.05	<0.05	<0.05	<0.05
苊	Acenaphthene	208-96-8	0.05	mg/kg	<0.05	<0.05	<0.05	<0.05	<0.05
二氢苊	Dihydrophenanthrene	83-32-9	0.05	mg/kg	<0.05	<0.05	<0.05	<0.05	<0.05
芴	Fluorene	86-73-7	0.05	mg/kg	<0.05	<0.05	<0.05	<0.05	<0.05
菲	Phenanthrene	85-01-8	0.05	mg/kg	<0.05	<0.05	<0.05	<0.05	<0.05
蒽	Anthracene	120-12-7	0.05	mg/kg	<0.05	<0.05	<0.05	<0.05	<0.05
荧蒽	Fluoranthene	206-44-0	0.05	mg/kg	<0.05	<0.05	<0.05	<0.05	<0.05
芘	Pyrene	129-00-0	0.05	mg/kg	<0.05	<0.05	<0.05	<0.05	<0.05
苯并(a)蒽	Benzo(a)anthracene	56-55-3	0.05	mg/kg	<0.05	<0.05	<0.05	<0.05	<0.05
屈	Chrysene	218-01-9	0.05	mg/kg	<0.05	<0.05	<0.05	<0.05	<0.05
苯并(k)荧蒽	Benzo (k) fluoranthene	207-08-9	0.05	mg/kg	<0.05	<0.05	<0.05	<0.05	<0.05
苯并(b)荧蒽	Benzo (b) fluoranthene	205-99-2	0.05	mg/kg	<0.05	<0.05	<0.05	<0.05	<0.05
苯并(a)芘	Benzo (a) pyrene	50-32-8	0.05	mg/kg	<0.05	<0.05	<0.05	<0.05	<0.05
茚并(1,2,3-cd)芘	Indeno (1,2,3-cd) pyrene	193-39-5	0.05	mg/kg	<0.05	<0.05	<0.05	<0.05	<0.05
二苯并(a,h)蒽	Dibenzo (a, h) anthracene	53-70-3	0.05	mg/kg	<0.05	<0.05	<0.05	<0.05	<0.05
苯并(g,h,i)苝	Benzo (g, h, i) perylene	191-24-2	0.05	mg/kg	<0.05	<0.05	<0.05	<0.05	<0.05

Sample status: Plant sample		Sample number<br>CAS	LOR	unit	SH110051<br>6-006	SH110051<br>6-007	SH110051<br>6-008	SH110051<br>6-009	SH110051<br>6-010
**多环芳烃 (Polycyclic aromatic hydrocarbons， PAHs)**									
萘	Naphthalene	91-20-3	0.05	mg/kg	<0.05	<0.05	<0.05	<0.05	<0.05
苊	Acenaphthene	208-96-8	0.05	mg/kg	<0.05	<0.05	<0.05	<0.05	<0.05
二氢苊	Dihydrophenanthrene	83-32-9	0.05	mg/kg	<0.05	<0.05	<0.05	<0.05	<0.05
芴	Fluorene	86-73-7	0.05	mg/kg	<0.05	<0.05	<0.05	<0.05	<0.05
菲	Phenanthrene	85-01-8	0.05	mg/kg	<0.05	<0.05	**0.06**	<0.05	<0.05
蒽	Anthracene	120-12-7	0.05	mg/kg	<0.05	<0.05	<0.05	<0.05	<0.05
荧蒽	Fluoranthene	206-44-0	0.05	mg/kg	<0.05	<0.05	<0.05	<0.05	**0.06**
芘	Pyrene	129-00-0	0.05	mg/kg	<0.05	**0.10**	**0.29**	<0.05	**0.08**
苯并(a)蒽	Benzo(a)anthracene	56-55-3	0.05	mg/kg	<0.05	<0.05	<0.05	<0.05	**0.07**
屈	Chrysene	218-01-9	0.05	mg/kg	<0.05	**0.30**	**1.17**	**0.22**	**0.68**
苯并(k)荧蒽	Benzo (k) fluoranthene	207-08-9	0.05	mg/kg	<0.05	<0.05	**0.20**	<0.05	**0.07**
苯并(b)荧蒽	Benzo (b) fluoranthene	205-99-2	0.05	mg/kg	<0.05	<0.05	<0.05	<0.05	<0.05
苯并(a)芘	Benzo (a) pyrene	50-32-8	0.05	mg/kg	<0.05	<0.05	**1.25**	<0.05	<0.05
茚并(1,2,3-cd)芘	Indeno (1,2,3-cd) pyrene	193-39-5	0.05	mg/kg	<0.05	<0.05	<0.05	<0.05	<0.05
二苯并(a,h)蒽	Dibenzo (a, h) anthracene	53-70-3	0.05	mg/kg	<0.05	<0.05	<0.05	<0.05	<0.05
苯并(g,h,i)苝	Benzo (g, h, i) perylene	191-24-2	0.05	mg/kg	<0.05	<0.05	<0.05	<0.05	<0.05

Sample status: Plant sample		Sample number<br>CAS	LOR	unit	SH110051<br>6-011	SH110051<br>6-012	SH110051<br>6-013	SH110051<br>6-014	SH110051<br>6-015
**多环芳烃 (Polycyclic aromatic hydrocarbons， PAHs)**									
萘	Naphthalene	91-20-3	0.05	mg/kg	<0.05	<0.05	<0.05	<0.05	<0.05
苊	Acenaphthene	208-96-8	0.05	mg/kg	<0.05	<0.05	<0.05	<0.05	<0.05
二氢苊	Dihydrophenanthrene	83-32-9	0.05	mg/kg	<0.05	<0.05	<0.05	<0.05	<0.05
芴	Fluorene	86-73-7	0.05	mg/kg	<0.05	<0.05	<0.05	<0.05	<0.05
菲	Phenanthrene	85-01-8	0.05	mg/kg	<0.05	<0.05	<0.05	**0.05**	<0.05
蒽	Anthracene	120-12-7	0.05	mg/kg	<0.05	<0.05	<0.05	<0.05	<0.05
荧蒽	Fluoranthene	206-44-0	0.05	mg/kg	<0.05	<0.05	<0.05	**0.07**	<0.05
芘	Pyrene	129-00-0	0.05	mg/kg	**0.08**	**0.07**	<0.05	**0.16**	<0.05
苯并(a)蒽	Benzo(a)anthracene	56-55-3	0.05	mg/kg	<0.05	<0.05	<0.05	**0.13**	<0.05
屈	Chrysene	218-01-9	0.05	mg/kg	<0.05	<0.05	**0.64**	**0.72**	**0.12**
苯并(k)荧蒽	Benzo (k) fluoranthene	207-08-9	0.05	mg/kg	<0.05	<0.05	<0.05	**0.10**	<0.05
苯并(b)荧蒽	Benzo (b) fluoranthene	205-99-2	0.05	mg/kg	<0.05	<0.05	<0.05	<0.05	<0.05
苯并(a)芘	Benzo (a) pyrene	50-32-8	0.05	mg/kg	<0.05	<0.05	<0.05	<0.05	<0.05
茚并(1,2,3-cd)芘	Indeno (1,2,3-cd) pyrene	193-39-5	0.05	mg/kg	<0.05	<0.05	<0.05	<0.05	<0.05
二苯并(a,h)蒽	Dibenzo (a, h) anthracene	53-70-3	0.05	mg/kg	<0.05	<0.05	<0.05	<0.05	<0.05
苯并(g,h,i)苝	Benzo (g, h, i) perylene	191-24-2	0.05	mg/kg	<0.05	<0.05	<0.05	<0.05	<0.05

Sample status: Plant sample		Sample number CAS	LOR	unit	SH110051 6-016	SH110051 6-017	SH110051 6-018	SH110051 6-019	SH110051 6-020
**多环芳烃 (Polycyclic aromatic hydrocarbons， PAHs)**									
萘	Naphthalene	91-20-3	0.05	mg/kg	<0.05	<0.05	<0.25	<0.05	<0.05
苊	Acenaphthene	208-96-8	0.05	mg/kg	<0.05	<0.05	<0.25	<0.05	<0.05
二氢苊	Dihydrophenanthrene	83-32-9	0.05	mg/kg	<0.05	<0.05	<0.25	<0.05	<0.05
芴	Fluorene	86-73-7	0.05	mg/kg	<0.05	<0.05	<0.25	<0.05	<0.05
菲	Phenanthrene	85-01-8	0.05	mg/kg	<0.05	<0.05	<0.25	<0.05	<0.05
蒽	Anthracene	120-12-7	0.05	mg/kg	<0.05	<0.05	<0.25	<0.05	<0.05
荧蒽	Fluoranthene	206-44-0	0.05	mg/kg	<0.05	<0.05	<0.25	**0.06**	**0.12**
芘	Pyrene	129-00-0	0.05	mg/kg	<0.05	<0.05	<0.25	**0.08**	**0.18**
苯并(a)蒽	Benzo(a)anthracene	56-55-3	0.05	mg/kg	<0.05	<0.05	<0.25	<0.05	<0.05
屈	Chrysene	218-01-9	0.05	mg/kg	**0.12**	<0.05	<0.25	**0.14**	**0.24**
苯并(k)荧蒽	Benzo (k) fluoranthene	207-08-9	0.05	mg/kg	<0.05	<0.05	<0.25	<0.05	<0.05
苯并(b)荧蒽	Benzo (b) fluoranthene	205-99-2	0.05	mg/kg	<0.05	<0.05	<0.25	<0.05	<0.05
苯并(a)芘	Benzo (a) pyrene	50-32-8	0.05	mg/kg	<0.05	<0.05	<0.25	<0.05	<0.05
茚并(1,2,3-cd)芘	Indeno (1,2,3-cd) pyrene	193-39-5	0.05	mg/kg	<0.05	<0.05	<0.25	<0.05	<0.05
二苯并(a,h)蒽	Dibenzo (a, h) anthracene	53-70-3	0.05	mg/kg	<0.05	<0.05	<0.25	<0.05	<0.05
苯并(g,h,i)苝	Benzo (g, h, i) perylene	191-24-2	0.05	mg/kg	<0.05	<0.05	<0.25	<0.05	<0.05

Sample status: Plant sample		Sample number CAS	LOR	unit	SH110051 6-021	SH110051 6-022	SH110051 6-023	SH110051 6-024	SH110051 6-025
**多环芳烃 (Polycyclic aromatic hydrocarbons， PAHs)**									
萘	Naphthalene	91-20-3	0.05	mg/kg	<0.05	<0.25	<0.05	<0.25	<0.05
苊	Acenaphthene	208-96-8	0.05	mg/kg	<0.05	<0.25	<0.05	<0.25	<0.05
二氢苊	Dihydrophenanthrene	83-32-9	0.05	mg/kg	<0.05	<0.25	<0.05	<0.25	<0.05
芴	Fluorene	86-73-7	0.05	mg/kg	<0.05	<0.25	<0.05	**0.28**	<0.05
菲	Phenanthrene	85-01-8	0.05	mg/kg	**0.05**	<0.25	**0.08**	**1.24**	<0.05
蒽	Anthracene	120-12-7	0.05	mg/kg	<0.05	<0.25	<0.05	<0.25	<0.05
荧蒽	Fluoranthene	206-44-0	0.05	mg/kg	<0.05	<0.25	<0.05	**0.84**	<0.05
芘	Pyrene	129-00-0	0.05	mg/kg	<0.05	<0.25	<0.05	**0.9**	<0.05
苯并(a)蒽	Benzo(a)anthracene	56-55-3	0.05	mg/kg	<0.05	<0.25	<0.05	**0.26**	<0.05
屈	Chrysene	218-01-9	0.05	mg/kg	0.05	<0.25	<0.05	**0.3**	0.73
苯并(k)荧蒽	Benzo (k) fluoranthene	207-08-9	0.05	mg/kg	<0.05	<0.25	<0.05	<0.25	<0.05
苯并(b)荧蒽	Benzo (b) fluoranthene	205-99-2	0.05	mg/kg	<0.05	<0.25	<0.05	<0.25	<0.05
苯并(a)芘	Benzo (a) pyrene	50-32-8	0.05	mg/kg	<0.05	<0.25	<0.05	<0.25	<0.05
茚并(1,2,3-cd)芘	Indeno (1,2,3-cd) pyrene	193-39-5	0.05	mg/kg	<0.05	<0.25	<0.05	<0.25	<0.05
二苯并(a,h)蒽	Dibenzo (a, h) anthracene	53-70-3	0.05	mg/kg	<0.05	<0.25	<0.05	<0.25	<0.05
苯并(g,h,i)苝	Benzo (g, h, i) perylene	191-24-2	0.05	mg/kg	<0.05	<0.25	<0.05	<0.25	<0.05

Sample status: Plant sample		Sample number CAS	LOR	unit	SH110051 6-026	SH110051 6-027	SH110051 6-028	SH110051 6-029	SH110051 6-030
**多环芳烃 (Polycyclic aromatic hydrocarbons， PAHs)**									
萘	Naphthalene	91-20-3	0.05	mg/kg	<0.05	<0.05	<0.05	<0.05	<0.05
苊	Acenaphthene	208-96-8	0.05	mg/kg	<0.05	<0.05	<0.05	<0.05	<0.05
二氢苊	Dihydrophenanthrene	83-32-9	0.05	mg/kg	<0.05	<0.05	<0.05	<0.05	<0.05
芴	Fluorene	86-73-7	0.05	mg/kg	<0.05	<0.05	<0.05	<0.05	<0.05
菲	Phenanthrene	85-01-8	0.05	mg/kg	**0.07**	<0.05	<0.05	<0.05	<0.05
蒽	Anthracene	120-12-7	0.05	mg/kg	<0.05	<0.05	<0.05	<0.05	<0.05
荧蒽	Fluoranthene	206-44-0	0.05	mg/kg	**0.13**	<0.05	<0.05	<0.05	<0.05
芘	Pyrene	129-00-0	0.05	mg/kg	**0.24**	<0.05	<0.05	<0.05	<0.05
苯并(a)蒽	Benzo(a)anthracene	56-55-3	0.05	mg/kg	**0.19**	<0.05	<0.05	<0.05	<0.05
屈	Chrysene	218-01-9	0.05	mg/kg	**1.65**	**0.1**	**0.06**	<0.05	<0.05
苯并(k)荧蒽	Benzo (k) fluoranthene	207-08-9	0.05	mg/kg	**0.13**	<0.05	<0.05	<0.05	<0.05
苯并(b)荧蒽	Benzo (b) fluoranthene	205-99-2	0.05	mg/kg	<0.05	<0.05	<0.05	<0.05	<0.05
苯并(a)芘	Benzo (a) pyrene	50-32-8	0.05	mg/kg	**0.05**	<0.05	<0.05	<0.05	<0.05
茚并(1,2,3-cd)芘	Indeno (1,2,3-cd) pyrene	193-39-5	0.05	mg/kg	<0.05	<0.05	<0.05	<0.05	<0.05
二苯并(a,h)蒽	Dibenzo (a, h) anthracene	53-70-3	0.05	mg/kg	<0.05	<0.05	<0.05	<0.05	<0.05
苯并(g,h,i)苝	Benzo (g, h, i) perylene	191-24-2	0.05	mg/kg	<0.05	<0.05	<0.05	<0.05	<0.05

Sample status: Plant sample		Sample number			SH110051 6-031	SH110051 6-032	SH110051 6-033	SH110051 6-034	SH110051 6-035
		CAS	LOR	unit					
**多环芳烃 (Polycyclic aromatic hydrocarbons， PAHs)**									
萘	Naphthalene	91-20-3	0.05	mg/kg	<0.05	<0.05	<0.05	<0.05	<0.05
苊	Acenaphthene	208-96-8	0.05	mg/kg	<0.05	<0.05	<0.05	<0.05	<0.05
二氢苊	Dihydrophenanthrene	83-32-9	0.05	mg/kg	<0.05	**0.07**	<0.05	<0.05	<0.05
芴	Fluorene	86-73-7	0.05	mg/kg	<0.05	**0.29**	<0.05	<0.05	<0.05
菲	Phenanthrene	85-01-8	0.05	mg/kg	<0.05	**0.31**	<0.05	<0.05	<0.05
蒽	Anthracene	120-12-7	0.05	mg/kg	<0.05	<0.05	<0.05	<0.05	<0.05
荧蒽	Fluoranthene	206-44-0	0.05	mg/kg	<0.05	**0.06**	<0.05	<0.05	<0.05
芘	Pyrene	129-00-0	0.05	mg/kg	<0.05	**0.06**	<0.05	<0.05	<0.05
苯并(a)蒽	Benzo(a)anthracene	56-55-3	0.05	mg/kg	<0.05	**0.1**	<0.05	<0.05	<0.05
屈	Chrysene	218-01-9	0.05	mg/kg	**0.13**	**0.9**	<0.05	**0.08**	<0.05
苯并(k)荧蒽	Benzo (k) fluoranthene	207-08-9	0.05	mg/kg	<0.05	**0.09**	<0.05	<0.05	<0.05
苯并(b)荧蒽	Benzo (b) fluoranthene	205-99-2	0.05	mg/kg	<0.05	<0.05	<0.05	<0.05	<0.05
苯并(a)芘	Benzo (a) pyrene	50-32-8	0.05	mg/kg	<0.05	<0.05	<0.05	**0.09**	<0.05
茚并(1,2,3-cd)芘	Indeno (1,2,3-cd) pyrene	193-39-5	0.05	mg/kg	<0.05	<0.05	<0.05	<0.05	<0.05
二苯并(a,h)蒽	Dibenzo (a, h) anthracene	53-70-3	0.05	mg/kg	<0.05	<0.05	<0.05	<0.05	<0.05
苯并(g,h,i)苝	Benzo (g, h, i) perylene	191-24-2	0.05	mg/kg	<0.05	<0.05	<0.05	<0.05	<0.05

Sample status: Plant sample		Sample number			SH110051 6-036
		CAS	LOR	unit	
**多环芳烃 (Polycyclic aromatic hydrocarbons， PAHs)**					
萘	Naphthalene	91-20-3	0.05	mg/kg	<0.05
苊	Acenaphthene	208-96-8	0.05	mg/kg	<0.05
二氢苊	Dihydrophenanthrene	83-32-9	0.05	mg/kg	<0.05
芴	Fluorene	86-73-7	0.05	mg/kg	**0.08**
菲	Phenanthrene	85-01-8	0.05	mg/kg	**0.06**
蒽	Anthracene	120-12-7	0.05	mg/kg	<0.05
荧蒽	Fluoranthene	206-44-0	0.05	mg/kg	<0.05
芘	Pyrene	129-00-0	0.05	mg/kg	**0.09**
苯并(a)蒽	Benzo(a)anthracene	56-55-3	0.05	mg/kg	<0.05
屈	Chrysene	218-01-9	0.05	mg/kg	**0.14**
苯并(k)荧蒽	Benzo (k) fluoranthene	207-08-9	0.05	mg/kg	<0.05
苯并(b)荧蒽	Benzo (b) fluoranthene	205-99-2	0.05	mg/kg	<0.05
苯并(a)芘	Benzo (a) pyrene	50-32-8	0.05	mg/kg	<0.05
茚并(1,2,3-cd)芘	Indeno (1,2,3-cd) pyrene	193-39-5	0.05	mg/kg	<0.05
二苯并(a,h)蒽	Dibenzo (a, h) anthracene	53-70-3	0.05	mg/kg	<0.05
苯并(g,h,i)苝	Benzo (g, h, i) perylene	191-24-2	0.05	mg/kg	<0.05
